# High-Energy Photon Attenuation Properties of Lead-Free and Self-Healing Poly (Vinyl Alcohol) (PVA) Hydrogels: Numerical Determination and Simulation

**DOI:** 10.3390/gels8040197

**Published:** 2022-03-22

**Authors:** Theerasarn Pianpanit, Kiadtisak Saenboonruang

**Affiliations:** 1 Department of Applied Radiation and Isotopes, Faculty of Science, Kasetsart University, Bangkok 10900, Thailand; fscitap@ku.ac.th; 2 Specialized Center of Rubber and Polymer Materials in Agriculture and Industry (RPM), Faculty of Science, Kasetsart University, Bangkok 10900, Thailand; 3 Special Research Unit of Radiation Technology for Advanced Materials, Faculty of Science, Kasetsart University, Bangkok 10900, Thailand

**Keywords:** PVA, Bi_2_O_3_, WO_3_, BaSO_4_, X-ray, gamma, shielding, XCOM, PHITS, simulation

## Abstract

This work numerically determined high-energy photon shielding properties of self-healing poly(vinyl alcohol) (PVA) hydrogels containing lead-free, heavy-metal compounds, namely, bismuth oxide (Bi_2_O_3_), tungsten oxide (WO_3_), and barium sulfate (BaSO_4_), through XCOM software packages. In order to understand the dependencies of the shielding properties of the hydrogels on filler contents and photon energies, the filler contents added to the hydrogels were varied from 0–40 wt.% and the photon energies were varied from 0.001–5 MeV. The results, which were verified for their reliability and correctness with those obtained from PHITS (Particle and Heavy Ion Transport code System), indicated that overall shielding performances, which included the mass attenuation coefficients (µ_m_), the linear attenuation coefficient (µ), the half-value layer (HVL), and the lead equivalence (Pb_eq_), of the hydrogels improved with increasing filler contents but generally decreased with increasing photon energies. Among the three compounds investigated in this work, Bi_2_O_3_/PVA hydrogels exhibited the highest photon attenuation capabilities, determined at the same filler content and photon energy, mainly due to its highest atomic number of Bi and the highest density of Bi_2_O_3_ in comparison with other elements and compounds. Furthermore, due to possible reduction in self-healing and mechanical properties of the hydrogels with excessive filler contents, the least content of fillers providing a 10-mm sample with the required Pb_eq_ value of 0.5 mmPb was investigated. The determination revealed that only the hydrogel containing at least 36 wt.% of Bi_2_O_3_ exhibited the Pb_eq_ values greater than 0.5 mmPb for all photon energies of 0.05, 0.08, and 0.1 MeV (common X-ray energies in general nuclear facilities). The overall outcomes of the work promisingly implied the potential of PVA hydrogels to be used as novel and potent X-ray and gamma shielding materials with the additional self-healing and nonlead properties.

## 1. Introduction

High-energy photons, especially X-rays and gamma rays, have been increasingly utilized in various applications, including material characterization [1,2,3], medical diagnostic and therapy [4,5], quality control in industrial products [6], plant mutation breeding [7], and national security [8]. Despite their great benefits, excessive exposures to X-rays and gamma rays pose serious biological effects on human populations, which could lead to a reduction in the immune system, the initiation of brain cancers, and the increase in mutation rates [9,10,11]. To cope with possible radiation illnesses and other side effects, a concept for radiation safety, namely, “As Low As Reasonably Achievable” or “ALARA”, consisting of the management of (1) exposure time, (2) distance between radiation sources and users, and (3) utilization of sufficient and appropriate shielding equipment, must be strictly practiced in all nuclear and radiation facilities [12].

Specifically for users who are required to work in a proximity to radiation sources for extended time periods, utilization of effective shielding equipment has become a necessity to prevent the users and the public from excessive exposure to radiations. In the case of X-rays and gamma rays, shielding equipment mostly relies on the use of heavy metals, especially lead (Pb) and lead oxide (PbO) [13], mainly due to their relatively higher interaction probabilities between the incident photons and Pb atoms, which significantly improved photon attenuation capabilities of the materials/composites as well as their economical availability [14]. Nonetheless, the toxicity of Pb has raised serious safety concerns as excessive exposure to Pb potentially leads to an increase in blood pressure, slow nerve conduction, fatigue, drowsiness, fertility disorders, encephalopathy, and death [15]. Furthermore, the spread of Pb in water resources and forests could negatively affect animals and plants. As a result, significant efforts to replace the toxic Pb with safer compounds have been emphasized and discussed in recent years. Among several potential candidates, bismuth oxide (Bi_2_O_3_), tungsten oxide (WO_3_), and barium sulfate (BaSO_4_) have shown promising possibilities to serve for such purposes due to the high atomic numbers (Z) of Bi, W, and Ba (Z = 83, 74, and 56, respectively) as well as their high densities (ρ = 8.9, 7.16, and 4.5 g/cm^3^, respectively), resulting in substantial enhancement of photon attenuation after being added to the main matrix [16]. For instance, Poltabtim et al. reported that natural rubber (NR) composites containing Bi_2_O_3_ numerically exhibited comparable X-ray and gamma shielding properties as those containing Pb; for instance, the µ_m_ values of Pb/NR and Bi_2_O_3_/NR composites were 0.094 and 0.092 cm^2^/g, respectively, determined at the filler content of 80 phr and the photon energy of 0.662 MeV) [17]. Maleksadeh et al. also confirmed the usability of WO_3_ and BaSO_4_ as Pb alternatives, evidenced by just a slight decrease in the attenuation capabilities of silicone-based composites containing WO_3_ and BaSO_4_ in comparison with those containing PbO (determined at the same filler content and photon energy) [18]. These reports clearly implied that Bi_2_O_3_, WO_3_, and BaSO_4_ could be utilized as safe and effective alternatives to Pb and Pb compounds in the production of high-energy photon equipment.

Additionally, the types of the main matrix used for the production of the shielding materials are one of key factors that could define other important characteristics and properties. For instance, materials based on polyethylene (PE) and polyvinyl chloride (PVC) such as Bi_2_O_3_/UHMWPE and Bi_2_O_3_/wood/PVC composites exhibited exceptional strength and rigidity, which were suitable for use as construction parts or movable equipment in nuclear facilities [19,20]. On the other hand, materials based on natural rubber (NR) and synthetic rubber (SR) such as Bi_2_O_3_/NR, WO_3_/NR, and Bi_2_O_3_/EPDM composites could be used as personal protective equipment (PPE) and covers of transporting casks due to their exceptional flexibility and elongation at break [21,22,23]. Nonetheless, the mentioned materials had some limitations due to the lack of self-healing capabilities, which may result in increased costs and procedures needed for repairment, replacement, and waste management of the damaged products. To alleviate such drawbacks, materials based on the autonomously self-healing poly(vinyl alcohol) (PVA) hydrogels have been recently developed to be used as radiation shielding materials [24,25]. An example of some PVA-based shielding materials is nano-Bi_2_O_3_/PVA hydrogels, which could attenuate gamma rays having the energies of 1.17 MeV and 1.33 MeV by 35% and 30%, respectively, while providing the percentage of recoverable strength (%Recovery) of 88.6% after being brought together for just 1 min [26]. Furthermore, these developed Bi_2_O_3_/PVA hydrogels were also lead-free, which additionally improve the health safety of users and the public with respect to the toxic lead. Another use of self-healing PVA hydrogels is Sm_2_O_3_/PVA and Gd_2_O_3_/PVA composites for neutron attenuation, for which both 1-cm-thick PVA hydrogels containing either 10.5 wt.% of Sm_2_O_3_ or Gd_2_O_3_ could reduce the initial intensity of incident thermal neutrons by almost 70%, while providing the %Recovery up to 70% after being brought into contact for 6 h [27]. These self-healing abilities of PVA hydrogels were due to the capabilities of PVA polymer chains to diffuse across fractured surfaces and initiate hydrogen bonds between two polymers (without any external stimuli after the damage) [26]. These two examples clearly indicated the great potentials of utilizing PVA hydrogels as autonomously self-healing and effective radiation shielding materials.

Despite the promising self-healable capabilities of PVA hydrogels, the addition of fillers, especially radiation protective fillers that tend to improve shielding properties of the composites with their increasing contents, may result in the decrease of %Recovery and, thus, the self-healing capabilities. This limitation was evidenced by the reports of Tiamduangtawan et al., which indicated that the increase in nano-Bi_2_O_3_ contents from 0 to 20 and 40 wt.% lowered the %Recovery of the hydrogels from 96.3% to 95.9% and 88.6%, respectively, while the increases in the Sm_2_O_3_ and Gd_2_O_3_ contents from 0 to 10.5 wt.% decreased the %Recovery of the self-healed hydrogels from 85% in pristine samples to ~65% and ~60%, respectively [26,27]. These negative relationships between filler contents and %Recovery were mainly due to increases in the cross-linking network of the PVA chains from physical interactions between the hydroxyl groups of PVA and the fillers, leading to the increase in overall crystallinity of the hydrogels that subsequently obstructed the chain diffusion and the initiation of self-healing mechanisms [24,26]. As a result, due to the competing roles of the fillers in enhancing photon attenuation capabilities and obstructing self-healing properties of the hydrogels, the least filler contents that provide the materials with sufficient photon shielding, while preserving the self-healing capabilities of the hydrogels, must be thoroughly determined.

As aforementioned, this work aimed to numerically determine high-energy photon shielding properties, which included the mass attenuation coefficient (µ_m_), the linear attenuation coefficient (µ), and the half-value layer (HVL), of PVA hydrogels containing either Bi_2_O_3_, WO_3_, or BaSO_4_ using an online software package, namely, XCOM [17,28,29]. In order to verify results from XCOM for further investigation, the results were compared with those obtained from a Monte Carlo particle transport simulation code, namely, PHITS (Particle and Heavy Ion Transport code System) [30,31]. The contents of the fillers and the photon energies used for the determination were varied from 0–40 wt.% and 0.001–5 MeV, respectively. Furthermore, the least contents, which could be regarded as the recommended contents for each filler at the photon energies of 0.05, 0.08, and 0.1 MeV, were determined by comparing the values of lead equivalence (Pb_eq_) of the 1-cm-thick PVA hydrogels with the required Pb_eq_ value of 0.5 mmPb. The outcomes of this work should not only reveal the numerical effectiveness of PVA hydrogels in photon attenuation but also promote the additional advantages of PVA hydrogels, especially the self-healing capability, in the applications of radiation protection.

## 2. Determination of High-Energy Photon Shielding Properties Using XCOM and PHITS

### 2.1. Determination of Mass Attenuaiton Coefficients (µ_m_)

The values of µ_m_ (the fraction of photons attenuated by a homogeneous material per unit mass) for PVA hydrogels containing varying Bi_2_O_3_, WO_3_, and BaSO_4_ contents of 0–40 wt.% were numerically determined using the XCOM software. Based on our previous experimental report to produce self-healing hydrogels, the content of PVA was fixed at 20 wt.% and the content of water was varied as 80-X wt.%, where X is the content of Bi_2_O_3_, WO_3_, and BaSO_4_. The XCOM software was developed and provided by the National Institute of Standards and Technology (NIST) (Gaithersburg, MD, USA), for which the photon cross-section database used for the determination of µ_m_ was the NIST standard reference database 8 (XGAM), released in 2010. In order to understand the dependency of µ_m_ behaviors on the energies of incident photons, the values of µ_m_ were determined at varying photon energies from 0.001–5 MeV, with the inclusion of coherent scattering [32].

In order to increase the usability of the work, simple mathematical relationships between µ_m_ and filler content at the photon energies of 0.1, 0.5, 1, and 5 MeV were also determined using the form shown in Equation (1):

(1) $$\mu_{m}=Ax+B$$

where µ_m_ is the mass attenuation coefficient, *x* is the filler content, and A (B) is the mathematical constant, determined using the Microsoft Excel software package. These determined mathematical relationships would allow us to reliably predict the value of µ_m_ at any filler content. It should be noted that this relationship in Equation (1) was selected based on our previous work [29], which showed a strong linear relationship between the values of µ_m_ and filler contents.

### 2.2. Verification of the Results from XCOM with PHITS

In order to reliably progress to further steps, the verification of the µ_m_ values obtained from XCOM was conducted by comparing the values with those obtained from PHITS [33] at the filler contents of 10, 20, 30, and 40 wt.% and the photon energies of 0.1, 0.5, 1, and 5 MeV. To obtain the µ_m_ values from PHITS, the photon beam with a diameter of 1 mm was pointed directly at the center of the sample, which had the surface area of 20 cm × 20 cm and the thickness of either 1 mm (for 0.1-MeV photons) or 1 cm (for 0.5-, 1-, and 5-MeV photons). More details of the PHITS setup can be found elsewhere [29]. The percentage differences between the values of µ_m_ obtained from XCOM and PHITS for all filler contents and photon energies of interest were also determined, following Equation (2):

(2) $$\mathrm{Difference}\;\left(\%\right)=\frac{\left|\mu_{m,\mathrm{XCOM}}-\mu_{m,\mathrm{PHITS}}\right|}{\mu_{m,\mathrm{XCOM}}}\times 100\%$$

where Difference (%) is the percentage difference between the values of µ_m_ obtained from XCOM and PHITS, µ_m,XCOM_ is the mass attenuation coefficient obtained from XCOM, and µ_m,PHITS_ is the mass attenuation coefficient obtained from PHITS. It is notable to point out that the considerably larger surface area of the sample in comparison with the diameter of the photon beam was to minimize the underestimated values of µ_m_ due to buildup effects [34]. 

### 2.3. Determination of Linear Attenuation Coefficient (µ) and Half-Value Layer (HVL) 

The values of µ (the fraction of photons attenuated by a material per unit length) and HVL (the required thickness of a material that could attenuate the intensity of incident photons by 50%) were calculated from the obtained values of µ_m_ in Section 2.1, following Equations (3) and (4), respectively:

(3) $$\mu ={\;\mu }_{m}\times \rho$$

(4) $$\mathrm{HVL}=\frac{\ln\left(2\right)}{\mu }$$

where *ρ* is the density of the PVA hydrogels, calculated from Equation (5):

(5) $$\rho =\frac{100}{\frac{C_{PVA}}{\rho_{PVA}}+\frac{C_{water}}{\rho_{water}}+\frac{C_{F}}{\rho_{F}}}\;$$

where *ρ_PVA_*, *ρ_water_*, and *ρ_F_* are the densities of PVA, water, and filler, respectively, and *C_PVA_*, *C_water_*, and *C_F_* are the contents of PVA, water, and filler, respectively. The values of ρ for individual compounds used for the determination of hydrogel densities are shown in Table 1.

Similar to µ_m_, mathematical relationships between µ (HVL) and filler content at the photon energies of 0.1, 0.5, 1, and 5 MeV were also determined, for which the forms for µ and HVL determination follow Equations (6) and (7), respectively:

(6) $$\mu =Ax^{2}+Bx+C$$

(7) $$\mathrm{HVL}=Ax^{5}+Bx^{4}+Cx^{3}+Dx^{2}+Ex+F$$

where A, B, C, D, E, and F are mathematical constants determined using a trendline function available in in the Microsoft Excel software package and *x* is the filler content. It should be noted that the forms of Equations (6) and (7) were selected due to their values of R^2^ (the correlation variance) being closest to 1 in comparison with those from other mathematical relationships.

### 2.4. Determination of Lead Equivalence (Pb_eq_)

The Pb_eq_ values of the Bi_2_O_3_/PVA, WO_3_/PVA, and BaSO_4_/PVA hydrogels at the photon energies of 0.05, 0.08, and 0.1 MeV were determined using Equation (8):

(8) $$\mu_{\mathrm{Pb}}{\mathrm{Pb}}_{\mathrm{eq}}=\mu_{\mathrm{PVA}}x$$

where µ_Pb_ is the mass attenuation coefficient of Pb; µ_PVA_ is the mass attenuation coefficient of Bi_2_O_3_/PVA, WO_3_/PVA, or BaSO_4_/PVA hydrogels; Pb_eq_ is the Pb equivalence (in mmPb); and *x* is the thickness of the sample, which was fixed at 10 mm for this work. The µ_Pb_ values used for the calculation of Pb_eq_ at the photon energies of 0.05, 0.08, and 0.1 MeV were 91.28, 27.46, and 62.98 cm^2^/g, respectively [26]. It is noteworthy that the selected photon energies for the determination of Pb_eq_ were based on the common X-ray energy ranges in nuclear facilities, for which the developed PVA hydrogels could be utilized as movable panels or PPE.

## 3. Results and Discussion

### 3.1. Mass Attenuation Coefficient (µ_m_)

**Figure 1:**
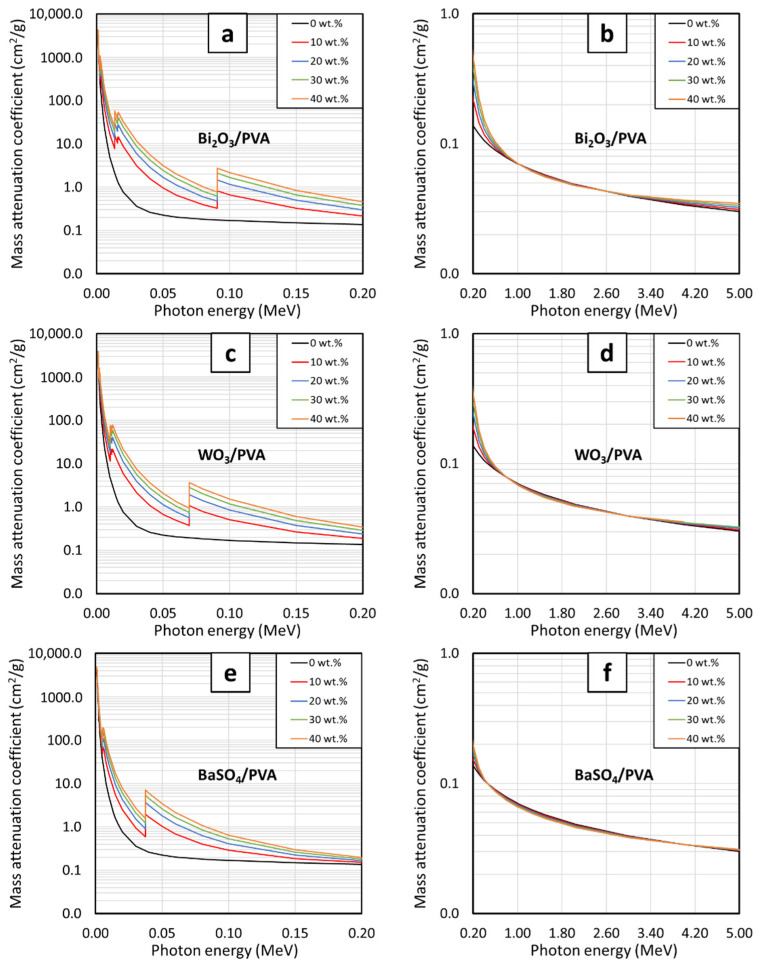
The µ_m_ values of (**a**,**b**) Bi_2_O_3_/PVA, (**c**,**d**) WO_3_/PVA, and (**e**,**f**) BaSO_4_/PVA hydrogels containing varying filler contents of 0, 10, 20, 30, and 40 wt.%, determined at photon energies of (**a**,**c**,**e**) 0.001–0.2 MeV and (**b**,**d**,**f**) 0.2–5 MeV using XCOM.

**Figure 2:**
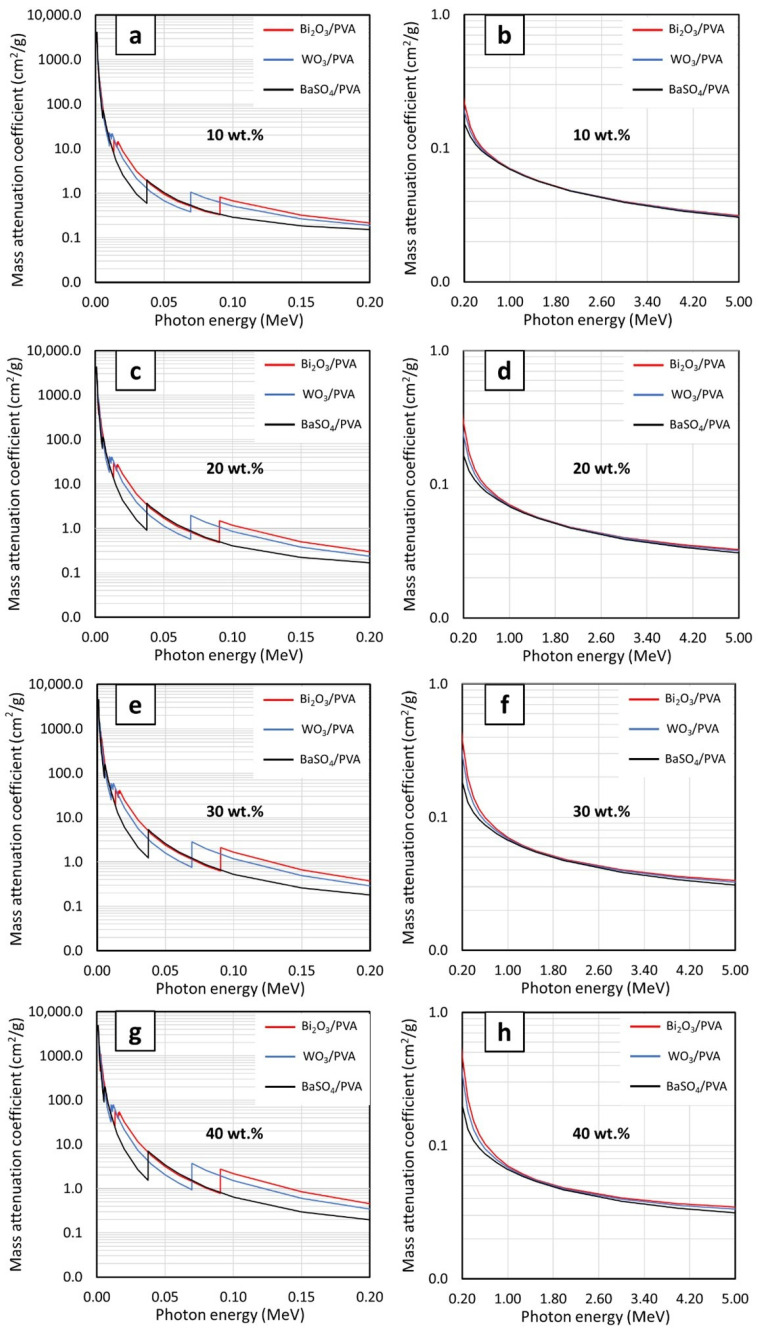
The µ_m_ values of Bi_2_O_3_/PVA, WO_3_/PVA, and BaSO_4_/PVA hydrogels containing filler contents of (**a**,**b**) 10 wt.%, (**c**,**d**) 20 wt.%, (**e**,**f**) 30 wt.%, and (**g**,**h**) 40 wt.%, determined at photon energies of (**a**,**c**,**e**,**g**) 0.001–0.2 MeV and (**b**,**d**,**f**,**h**) 0.2–5 MeV using XCOM.

The behaviors of µ_m_ for PVA hydrogels containing varying contents of Bi_2_O_3_, WO_3_, and BaSO_4_ at photon energies of 0.001–5 MeV are shown in Figure 1 and Figure 2. Figure 1, which compares the µ_m_ values of PVA hydrogels having the same filler type but varying filler contents of 10, 20, 30, and 40 wt.%, indicates that µ_m_ values generally increased with increasing filler contents but decreased with increasing photon energies. The effects of filler contents on the enhancement of photon attenuation capabilities were mainly due to their relatively higher atomic numbers (Z) of Bi, W, and Ba (Z = 83, 74, and 56, respectively) in the fillers compared with those of H, C, and O (Z = 1, 6, and 8, respectively) in the PVA and water as well as their much higher densities of Bi_2_O_3_, WO_3_, and BaSO_4_ in comparison with those of PVA and water (Table 1), resulting in enhanced interaction probabilities between the incident photons and Bi_2_O_3_, WO_3_, or BaSO_4_ fillers in PVA hydrogels. It is notable that the effects of fillers on the enhancement of photon shielding properties were more pronounced at lower photon energies (0.001–0.5 MeV) than those at higher energies (0.5–5 MeV). This was because the interactions of photons with the hydrogels at lower energies were dominated by the process of photoelectric absorption, for which its interaction probability, i.e., its photoelectric cross section (σ_pe_), greatly depends on photon energies and atomic numbers of the material, as shown in Equation (9):

(9) $$\sigma_{\mathrm{pe}}\propto \frac{Z^{n}}{{\left(h\nu \right)}^{3}}$$

where σ_pe_ is the photoelectric cross section (a nuclear quantity representing the interaction probabilities of an element or a material with incident radiation through the photoelectric absorption), Z is the atomic number of the element contained in the material, h is a Planck’s constant, and ν is the frequency of the incident photon, which is directly proportional to the photon energy (E = hν) [29,36]. As implied in Equation (9), σ_pe_ of a material and subsequently the ability to attenuate photons would be substantially enhanced when more heavy elements (Bi, W, and Ba in this case) were added to the hydrogels, which were clearly illustrated in Figure 1a,c,d.

On the other hand, Equation (9) depicts that the µ_m_ values would rapidly decrease with increasing photon frequencies (energies). This behavior was observed due to the inverse relationship between σ_pe_ and ν (σ_pe_ ∝ 1/ν^3^) that results in sharp declines of interaction probabilities between the incident photons and the hydrogels at higher photon energies, which subsequently reduced their attenuation capabilities. It should be noted that the µ_m_ values for all hydrogels shown in Figure 1b,d,f were very close to each other (regardless of their filler contents), especially at the photon energies of 0.5–3 MeV. This was due to the diminishing roles of photoelectric absorption at these energies and the change in the dominant interaction mechanism from photoelectric absorption to a less effective attenuation mechanism, namely, Compton scattering. The dependency of the Compton scattering cross section (σ_comp_) on the characteristics of the material could be described as shown in Equation (10):

(10) $$\sigma_{comp}\propto \;\frac{1}{n_{e}}\;$$

where n_e_ is the electron density of the material [29,37]. 

However, as the photon energies further increased to higher than 3 MeV, increases in filler contents started to affect the µ_m_ values again. This was mainly due to the initiation of another important interaction of the photon, namely, the pair production, which starts at the photon energies greater than 1.022 MeV and becomes the dominant interaction mechanism at the energies around 3 MeV. The pair production probabilities, which are represented by the values of its cross section (σ_pp_), are directly proportional to the square of atomic number (Z), as shown in Equation (11):

(11) $$\sigma_{\mathrm{pp}}\propto Z^{2}$$

According to Equation (11), it implies that the µ_m_ values of the hydrogels are enhanced as more fillers are added to the hydrogels, resulting in more pronounced differences in µ_m_ values, as observed in Figure 1b,d,f (after 3 MeV) [17,38].

**Figure 3:**
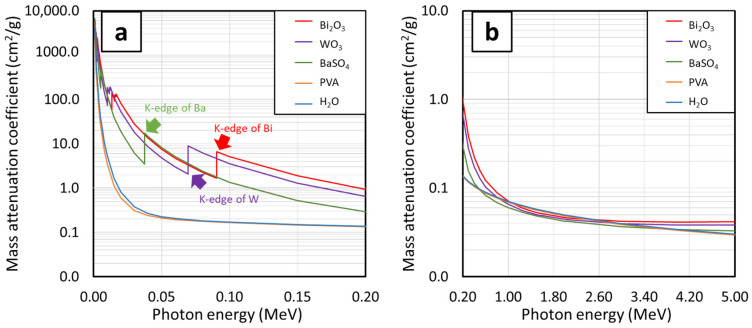
The µ_m_ values of Bi_2_O_3_, WO_3_, BaSO_4_, PVA, and H_2_O showing K-edge and L-edge behaviors of Bi, W, and Ba at photon energies of (**a**) 0.001–0.2 MeV and (**b**) 0.2–5 MeV using XCOM.

Figure 2a–d, which compares µ_m_ behaviors of PVA composites containing Bi_2_O_3_, WO_3_, or BaSO_4_ at the same filler contents of 10, 20, 30, and 40 wt.%, respectively, reveals that Bi_2_O_3_/PVA hydrogels generally exhibited the highest µ_m_ values (determined at the same filler content and photon energy), except at the photon energies of 0.010–0.012 MeV and 0.069–0.090 MeV, for which WO_3_/PVA hydrogels had higher µ_m_ values than Bi_2_O_3_/PVA and BaSO_4_/PVA hydrogels. The superior photon attenuation capabilities of Bi_2_O_3_/PVA hydrogels at most photon energies were observed due to their relatively higher atomic number and density than those of WO_3_, BaSO_4_, PVA, and water (Figure 3), leading to higher µ_m_ values for Bi_2_O_3_/PVA hydrogels. It is noteworthy that, at the photon energies of 0.010–0.012 MeV and 0.069–0.090 MeV, WO_3_/PVA hydrogels had uncharacteristically higher µ_m_ values than other hydrogels. This was due to the effects of W’s K-absorption edge (K-edge) and L-absorption edge (L-edge) that occurred at 0.069 MeV and 0.010–0.012 MeV, respectively, for which the σ_pe_ and, subsequently, the µ_m_ of WO_3_/PVA hydrogels abruptly increased at these particular energies [39].

**Figure 4:**
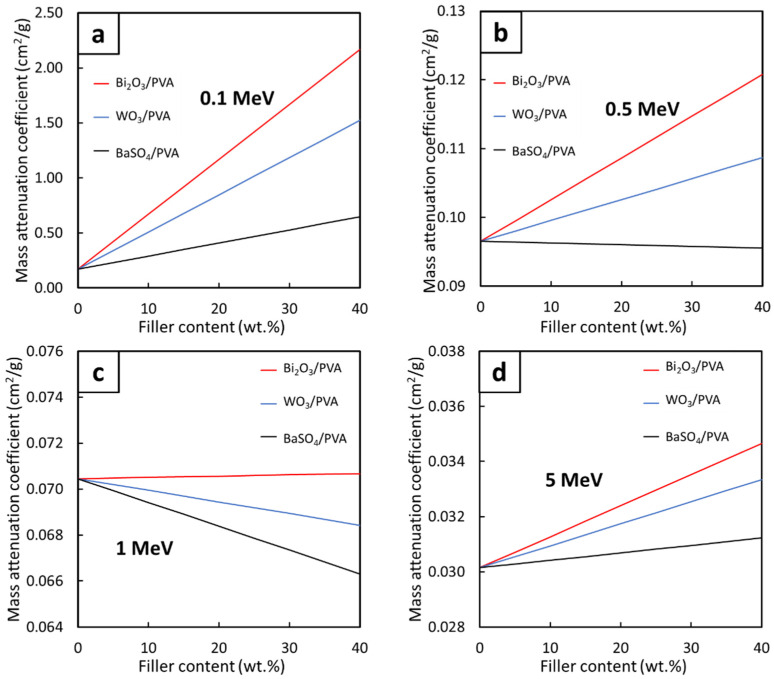
The µ_m_ values of Bi_2_O_3_/PVA, WO_3_/PVA, and BaSO_4_/PVA hydrogels containing varying filler contents of 0–40 wt.%, determined at photon energies of (**a**) 0.1 MeV, (**b**) 0.5 MeV, (**c**) 1 MeV, and (**d**) 5 MeV using XCOM.

Figure 4, which compares the µ_m_ values for the Bi_2_O_3_/PVA, WO_3_/PVA, and BaSO_4_/PVA hydrogels with varying filler contents of 0–40 wt.% determined at the photon energies of 0.1, 0.5, 1, and 5 MeV, reveals that strong linear correlations between the filler contents and the µ_m_ values were observed for all investigated energies. While most of the relationships were positively correlated (Figure 4a,d), mainly due to the roles of the fillers in photon attenuation through the dominant photoelectric absorption (Figure 4a) and the pair production (Figure 4d), some of the relationships such as those of BaSO_4_/PVA (Figure 4b,c) and WO_3_/PVA (Figure 4c) were found to be negatively correlated. These negative trends were observed because, at these particular photon energies, the pristine PVA hydrogels, which contain higher light-element (C, H, and O) contents, could better interact with the photons through the dominant Compton scattering than those of PVA hydrogels containing the fillers [26]. This phenomenon could be mathematically explained using Equation (10) as σ_comp_ is inversely proportional to the electron densities (n_e_) of the composites, implying that the pristine PVA hydrogels would have higher σ_comp_ and, consequently, slightly higher photon attenuation capabilities (Figure 3b).

In order to enable the ability to estimate the µ_m_ values for all filler contents at the photon energies of 0.1, 0.5, 1, and 5 MeV (Figure 4), mathematical equations with the form shown in Equation (1) were determined. The results, which are shown in Table 2, indicated that Bi_2_O_3_/PVA hydrogels exhibited the strongest correlations between the filler contents and the µ_m_ values, evidenced by the highest slopes (shown as the values of the constant A) for all photon energies. These were mainly due to the highest atomic number of Bi (Z = 83) in comparison with those of W (Z = 74) and Ba (Z = 56), which resulted in higher interaction probabilities and greater effects of the filler on the enhancement of photon shielding properties. It is notable that the constants A for all hydrogels at the photon energy of 0.1 MeV were relatively higher than those found at higher energies. This was due to a greater dependence of σ_pe_ values on the atomic number (Z) of the hydrogels (Equation (9)), for which the photoelectric absorption was the dominant photon interaction for the 0.1-MeV photons.

To verify the reliability and correctness of the numerical results obtained in this work, the results from XCOM were compared with those obtained from a Monte Carlo simulation code, PHITS. The filler contents used for the comparison were 10, 20, 30, and 40 wt.% (determined at the photon energies of 0.1, 0.5, 1, and 5 MeV). The results of the comparison as well as their percentage of difference (Difference (%)) are shown in Table 3, which indicates that the results obtained from the two methods were in good agreement, with the range and the average of the Difference (%) being 0.02–1.40% and 0.53%, respectively. Hence, based on the comparison, the results from XCOM could be reliably used in later determinations of µ, HVL, and Pb_eq_.

### 3.2. Linear Attenuation Coefficient (µ) and Half-Value Layer (HVL)

To determine the values of µ and HVL, the densities of all PVA hydrogels containing either Bi_2_O_3_, WO_3_, or BaSO_4_ were theoretically calculated based on Equation (5) and individual densities (Table 1). The results, which are shown in Table 4, indicated that the densities of PVA hydrogels increased with increasing filler content, mainly due to much higher densities of the fillers in comparison with those of PVA and water. Furthermore, the results suggested that Bi_2_O_3_/PVA hydrogels exhibited higher densities than those of WO_3_/PVA and BaSO_4_/PVA hydrogels at the same filler content. This behavior was observed due to relatively higher densities on Bi_2_O_3_ (Table 1).

**Figure 5:**
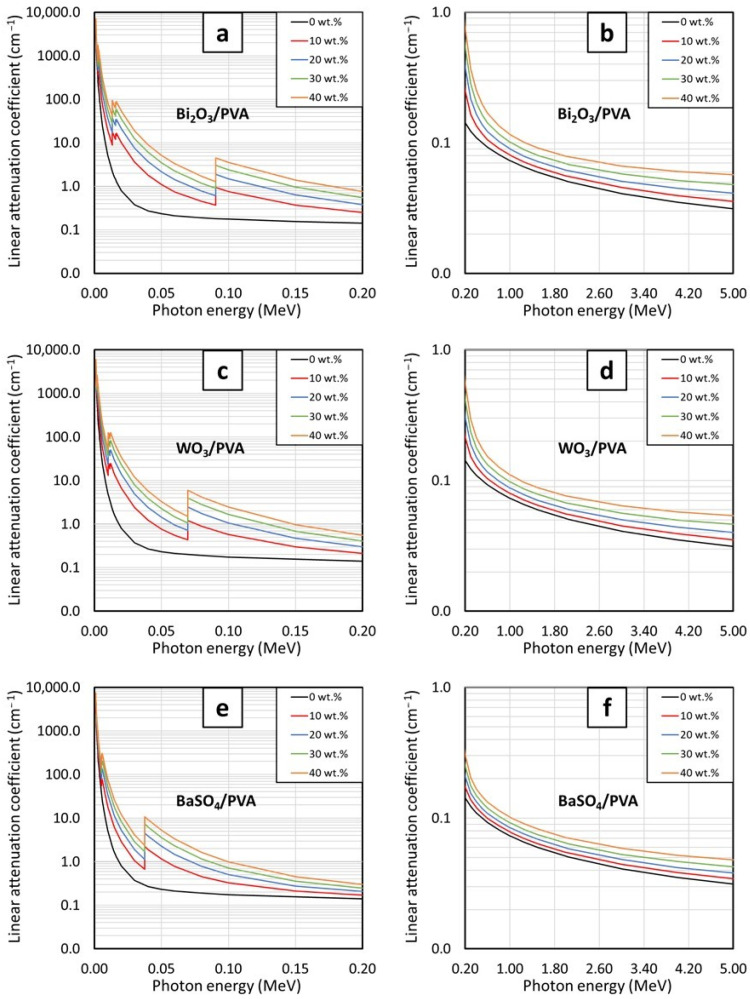
The µ values of (**a**,**b**) Bi_2_O_3_/PVA, (**c**,**d**) WO_3_/PVA, and (**e**,**f**) BaSO_4_/PVA hydrogels containing varying filler contents of 0, 10, 20, 30, and 40 wt.%, determined at photon energies of (**a**,**c**,**e**) 0.001–0.2 MeV and (**b**,**d**,**f**) 0.2–5 MeV using XCOM.

**Figure 6:**
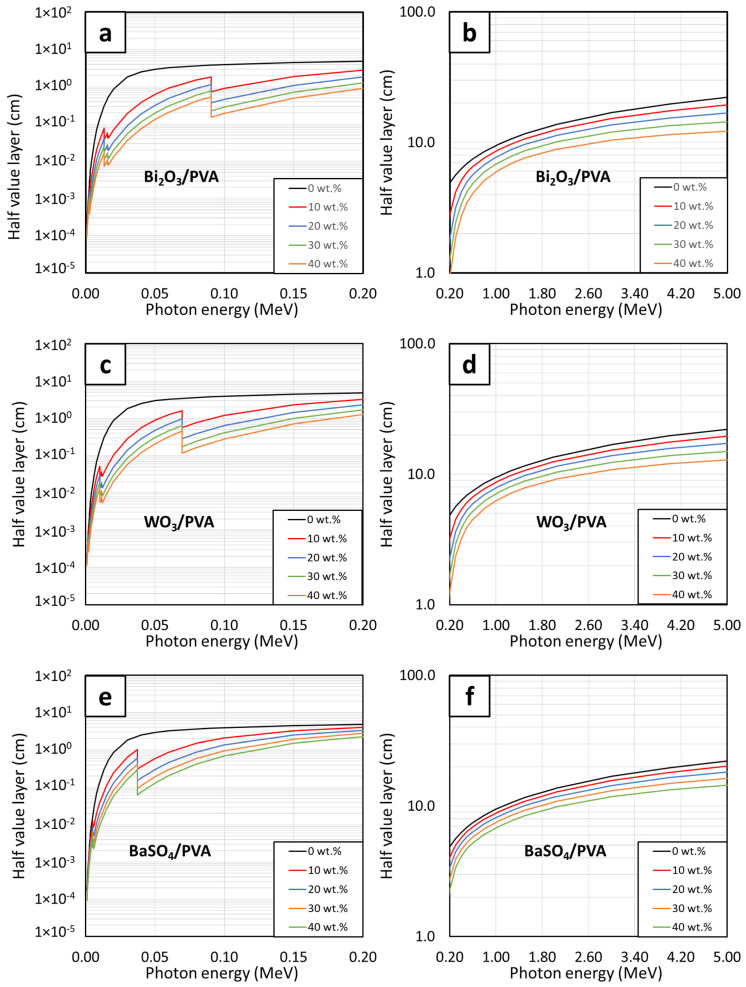
HVL values of (**a**,**b**) Bi_2_O_3_/PVA, (**c**,**d**) WO_3_/PVA, and (**e**,**f**) BaSO_4_/PVA hydrogels containing varying filler contents of 0, 10, 20, 30, and 40 wt.%, determined at photon energies of (**a**,**c**,**e**) 0.001–0.2 MeV and (**b**,**d**,**f**) 0.2–5 MeV using XCOM.

The linear attenuation coefficients (µ) and the half-value layer (HVL) of the PVA hydrogels, calculated using Equations (3) and (4), respectively, are shown in Figure 5 and Figure 6, respectively. The results indicated that, similarly to the behaviors of µ_m_, the values of µ and HVL generally improved with increasing filler contents, evidenced by the highest values of µ and the lowest values of HVL observed in the PVA hydrogels containing 40 wt.% of the fillers. In addition, the effects of additional filler contents on the µ and HVL values were found to be more pronounced than those observed in the case of µ_m_, evidenced by larger differences in the µ values for each 10 wt.% addition of the fillers. This behavior was observed due to the high densities of the PVA hydrogels (Table 4), which subsequently amplified the µ values of the hydrogels according to Equation (3).

**Figure 7:**
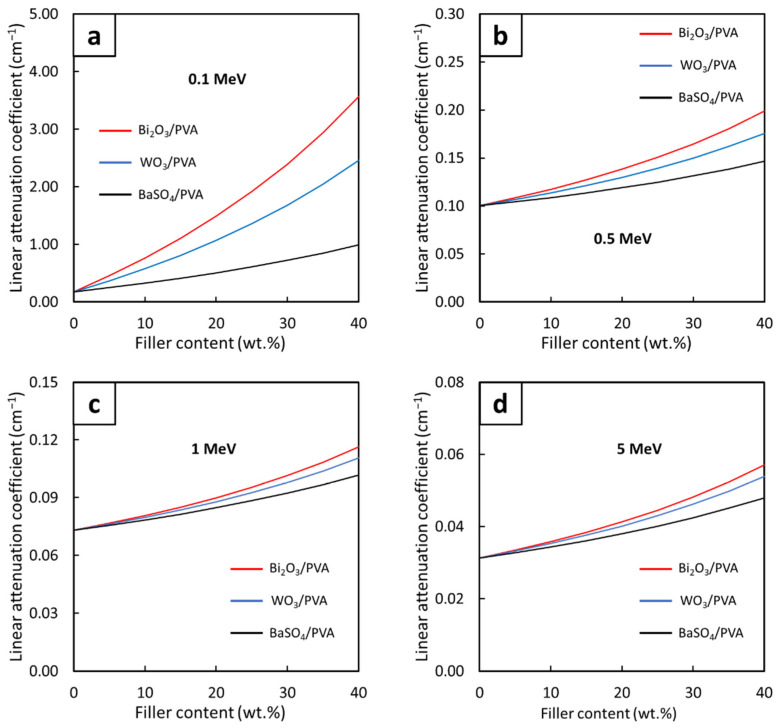
The µ values of Bi_2_O_3_/PVA, WO_3_/PVA, and BaSO_4_/PVA hydrogels containing varying filler contents of 0–40 wt.%, determined at photon energies of (**a**) 0.1 MeV, (**b**) 0.5 MeV, (**c**) 1 MeV, and (**d**) 5 MeV using XCOM.

**Figure 8:**
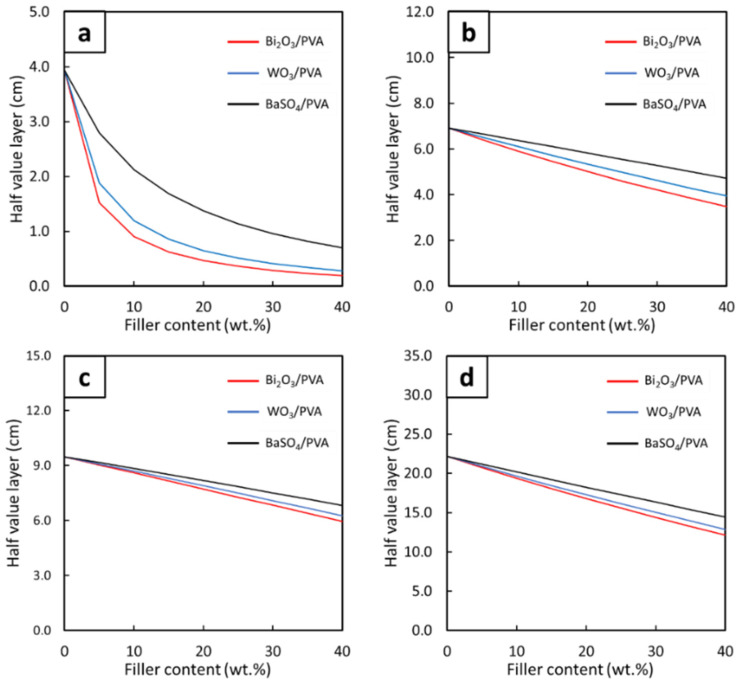
The HVL values of Bi_2_O_3_/PVA, WO_3_/PVA, and BaSO_4_/PVA hydrogels containing varying filler contents of 0–40 wt.%, determined at photon energies of (**a**) 0.1 MeV, (**b**) 0.5 MeV, (**c**) 1 MeV, and (**d**) 5 MeV using XCOM.

Figure 7 and Figure 8 showed correlations between the filler contents and the µ and HVL values, respectively, at the photon energies of 0.1, 0.5, 1, and 5 MeV. Both figures suggested that additional filler contents could increase (decrease) the values of µ (HVL) with greater effects than those observed in µ_m_, which was evidenced by the non-linear correlations between the filler contents and µ (HVL) values (Figure 7). Similar to µ_m_, mathematical relationships between the filler contents and the µ and HVL values at the photon energies of 0.1, 0.5, 1, and 5 MeV were determined based on Equations (6) and (7), respectively. The results, which are shown in Table 5 and Table 6, clearly showed that the addition of the fillers could improve photon attenuation capabilities of the hydrogels, as seen by the positive constants A in the case of µ for all photon energies and the negative constants A (E) in the case of HVL for the photon energy of 0.1 MeV (0.5, 1, and 5 MeV). Furthermore, the relatively greater effects of Bi_2_O_3_ on the enhancement of photon shielding properties in comparison with those of WO_3_ and BaSO_4_ were confirmed from this determination as the magnitudes of constants A in the case of Bi_2_O_3_/PVA hydrogels were the greatest among other hydrogels. 

### 3.3. Lead Equivalence (Pb_eq_)

The Pb_eq_ values of PVA hydrogels containing Bi_2_O_3_, WO_3_, or BaSO_4_ with the filler contents of 10, 20, 30, and 40 wt.% (determined at the photon energies of 0.05, 0.08, and 0.1 MeV) are shown in Table 7. The results indicated that the Pb_eq_ values for all hydrogels increased with increasing filler contents, which was consistent with the behaviors of µ_m_ and µ. Generally, the Bi_2_O_3_/PVA hydrogels exhibited higher Pb_eq_ values than WO_3_/PVA and BaSO_4_/PVA hydrogels, mostly due to their higher atomic number of Bi and higher density of Bi_2_O_3_, for which the highest Pb_eq_ values achieved in 40 wt.% Bi_2_O/PVA hydrogels were found to be 0.57, 0.61, and 0.57 mmPb at the photon energies of 0.05, 0.08, and 0.1 MeV, respectively. However, at the photon energy of 0.08 MeV, WO_3_/PVA hydrogels offered higher Pb_eq_ values than those of Bi_2_O_3_/PVA hydrogels, determined at the same filler content. This behavior was observed due to the effects of the K-absorption edge of W, which occurred at 0.069 MeV, that resulted in uncharacteristically high interaction probabilities between the incident photons and W atoms at the specific photon energy (Figure 3), leading to the Pb_eq_ values for 40 wt.% WO_3_/PVA hydrogels as high as 1.53 mmPb [39].

Although the requirements for the Pb_eq_ of shielding materials varied, depending on applications and photon energies, a common Pb_eq_ requirement for shielding equipment used in general nuclear facilities is to be at least 0.5 mmPb [40]. Hence, Table 7 suggests that Bi_2_O_3_/PVA hydrogels with the filler content of at least 36 wt.% (interpolated from the hydrogels with 30 wt.% and 40 wt.% of Bi_2_O_3_) were the recommended conditions for all investigated photon energies (0.05, 0.08, and 0.1 MeV). On the other hand, the results showed that WO_3_/PVA and BaSO_4_/PVA hydrogels required the filler contents beyond 40 wt.% at some photon energies (0.05 MeV for WO_3_/PVA hydrogels and 0.1 MeV for both WO_3_/PVA and BaSO_4_/PVA hydrogels) as their Pb_eq_ values were lower than the required 0.5 mmPb at the maximum filler content investigated in this work of 40 wt.%. Although 34 wt.% of Bi_2_O_3_ was sufficient for the photon attenuation at the energy of 0.08 MeV, the actual photon energies inside nuclear facilities were distributed as a spectrum; hence, 36 wt.% of Bi_2_O_3_ would be a better choice in order to cover all photon energies of concern. It should be noted that, since Pb_eq_ values depend on sample thickness, one could reduce the recommended filler contents for each photon energy by increasing sample thicknesses, which would also improve the recoverable strength of the self-healed hydrogels. Nonetheless, the choice of using thicker samples would be applicable only in applications that allowed or accepted thicker materials to serve their intended requirements.

Furthermore, based on our previous work, it could be implied that PVA hydrogels containing 36 wt.% of Bi_2_O_3_ would offer percentages of recoverable strength (%Recovery) of at least 75%, the initial tensile strengths of at least 219 MPa, and the elongation at break of at least 560%, for which the actual values depended on several factors such as the particle sizes of Bi_2_O_3_, a procedure to prepare the hydrogels, and the types of PVA granules [26]. Lastly, as the recommended contents of WO_3_ and BaSO_4_ were close to or beyond 40 wt.% and no actual experiment was conducted to confirm the producibility of such formulations, we positively believed that WO_3_/PVA and BaSO_4_/PVA hydrogels were producible as the %Recovery of 40 wt.% Bi_2_O_3_/PVA hydrogel was as high as 88.6%, which implied that 40 wt.% WO_3_/PVA and 40 wt.% BaSO_4_/PVA hydrogels should have similar or slightly lower mechanical properties and %Recovery in comparison with those of a 40 wt.% Bi_2_O_3_/PVA hydrogel. The similarity of the production and characteristics among Bi_2_O_3_, WO_3_, and BaSO_4_ fillers in radiation shielding materials was supported by previous works of Poltabtim et al. [16], Toyen et al. [23], and Abdolahzadeh et al. [41], who experimentally prepared Bi_2_O_3-_, WO_3-_, or BaSO_4-_filled EPDM, NR, and HDPE, respectively.

## 4. Conclusions

This work determined the numerical high-energy photon shielding properties of autonomously self-healing PVA hydrogels containing Bi_2_O_3_, WO_3_, or BaSO_4_ with varying filler contents of 0–40 wt.% using XCOM. The shielding properties investigated in this work consisted of µ_m_, µ, HVL, and Pb_eq_ at the photon energies of 0.001–5 MeV as well as the recommended filler contents, determined at the photon energies of 0.05, 0.08, and 0.1 MeV. The results, which were in good agreement with those obtained from PHITS, revealed that the values of µ_m_ and µ (HVL) increased (decreased) with increasing filler contents but decreased (increased) with photon energies. In addition, the results suggested that Bi_2_O_3_/PVA hydrogels generally attenuated photons with higher efficiencies than those of WO_3_/PVA and BaSO_4_ hydrogels at the same filler content and photon energy. Lastly, the determination of recommended filler contents showed that Bi_2_O_3_/PVA hydrogels with the filler content of 36 wt.% exhibited the Pb_eq_ values higher than the required 0.5 mmPb for all investigated photon energies, implying that the PVA hydrogels with at least 36 wt.% of Bi_2_O_3_ were suitable for use as flexible and self-healing X-ray and gamma shielding equipment in general nuclear facilities.

## Figures and Tables

**Table 1:** Individual densities of PVA, Bi_2_O_3_, WO_3_, BaSO_4_, and water used for the determination of densities for PVA hydrogels [16,26,35].

Matrix/Compound	Chemical Formula	Density (g/cm^3^)
Poly(vinyl alcohol)	[CH_2_CH(OH)]_n_	1.23
Bismuth oxide	Bi_2_O_3_	8.90
Tungsten oxide	WO_3_	7.16
Barium sulfate	BaSO_4_	4.50
Water	H_2_O	1.00

**Table 2:** Mathematical constants (A and B) for µ_m_ in the form µ_m_ = Ax + B (Equation (1)), determined from Figure 4.

Photon Energy (MeV)	Bi_2_O_3_		WO_3_		BaSO_4_	
	A	B	A	B	A	B
0.1	0.0499	0.1698	0.0338	0.1696	0.0119	0.1697
0.5	6.06 × 10^−4^	0.0965	3.05 × 10^−4^	0.0965	−2.49 × 10^−5^	0.0965
1.0	5.53 × 10^−6^	0.0705	−5.05 × 10^−5^	0.0705	−1.04 × 10^−4^	0.0705
5.0	1.13 × 10^−4^	0.0301	7.99 × 10^−5^	0.0301	2.73 × 10^−5^	0.0301

**Table 3:** Comparative µ_m_ values obtained from XCOM and PHITS for PVA hydrogels containing Bi_2_O_3_, WO_3_, and BaSO_4_ at varying photon energies of 0.1, 0.5, 1, and 5 MeV.

Filler	Photon Energy (MeV)	Content (wt.%)	µ (cm^−1^)		Difference (%)
			XCOM	PHITS	
Bi_2_O_3_	0.1	10	0.76547	0.76845	0.39%
		20	1.48776	1.48390	0.26%
		30	2.39405	2.39545	0.06%
		40	3.56517	3.55330	0.33%
	0.5	10	0.11741	0.11707	0.29%
		20	0.13833	0.13817	0.11%
		30	0.16473	0.16584	0.67%
		40	0.19883	0.19968	0.43%
	1.0	10	0.08069	0.08112	0.54%
		20	0.08988	0.09008	0.22%
		30	0.10142	0.10196	0.54%
		40	0.11632	0.11661	0.25%
	5.0	10	0.03578	0.03591	0.39%
		20	0.04127	0.04142	0.36%
		30	0.04815	0.04881	1.39%
		40	0.05703	0.05782	1.40%
WO_3_	0.1	10	0.57919	0.58535	1.06%
		20	1.06978	1.06957	0.02%
		30	1.68062	1.68674	0.36%
		40	2.46092	2.46264	0.07%
	0.5	10	0.11357	0.11371	0.13%
		20	0.12979	0.12937	0.32%
		30	0.14989	0.15053	0.43%
		40	0.17576	0.17692	0.66%
	1.0	10	0.07980	0.08048	0.86%
		20	0.08784	0.08811	0.31%
		30	0.09786	0.09827	0.42%
		40	0.11064	0.11102	0.34%
	5.0	10	0.03530	0.03552	0.64%
		20	0.04015	0.04029	0.36%
		30	0.04619	0.04661	0.92%
		40	0.05391	0.05460	1.28%
BaSO_4_	0.1	10	0.32651	0.32995	1.06%
		20	0.50555	0.51169	1.22%
		30	0.72278	0.73043	1.06%
		40	0.99203	0.99395	0.19%
	0.5	10	0.10879	0.10925	0.43%
		20	0.11896	0.11930	0.29%
		30	0.13131	0.13147	0.13%
		40	0.14660	0.14654	0.04%
	1.0	10	0.07846	0.07920	0.95%
		20	0.08473	0.08554	0.97%
		30	0.09235	0.09272	0.41%
		40	0.10178	0.10181	0.04%
	5.0	10	0.03438	0.03461	0.69%
		20	0.03803	0.0383	0.84%
		30	0.04245	0.04271	0.63%
		40	0.04795	0.04833	0.80%

**Table 4:** Densities of PVA hydrogels containing Bi_2_O_3_, WO_3_, and BaSO_4_ with varying contents of 0–40 wt.% (in 5 wt.% increments), calculated using Equation (5).

Content (wt.%)	Density (g/cm^3^)		
	Bi_2_O_3_	WO_3_	BaSO_4_
0	1.039	1.039	1.039
5	1.089	1.087	1.083
10	1.144	1.141	1.130
15	1.206	1.200	1.182
20	1.274	1.265	1.239
25	1.350	1.338	1.302
30	1.436	1.419	1.371
35	1.534	1.512	1.448
40	1.646	1.617	1.535

**Table 5:** Mathematical constants (A, B, and C) for µ in the form µ = A*x*^2^ + B*x* + C (Equation (6)), determined from Figure 7.

Photon Energy (MeV)	Bi_2_O_3_			WO_3_			BaSO_4_		
	A	B	C	A	B	C	A	B	C
0.1	9.58 × 10^−4^	4.52 × 10^−2^	1.98 × 10^−1^	6.23 × 10^−4^	3.15 × 10^−2^	1.90 × 10^−1^	1.97 × 10^−4^	1.23 × 10^−2^	1.80 × 10^−1^
0.5	2.79 × 10^−5^	1.31 × 10^−3^	1.01 × 10^−1^	2.06 × 10^−5^	1.04 × 10^−3^	1.01 × 10^−1^	1.12 × 10^−5^	7.00 × 10^−3^	1.00 × 10^−1^
1.0	1.22 × 10^−5^	5.76 × 10^−4^	7.35 × 10^−2^	1.02 × 10^−5^	5.17 × 10^−4^	7.34 × 10^−2^	6.89 × 10^−6^	4.32 × 10^−4^	7.33 × 10^−2^
5.0	7.28 × 10^−6^	3.43 × 10^−4^	3.15 × 10^−2^	6.17 × 10^−6^	3.11 × 10^−4^	3.14 × 10^−2^	4.03 × 10^−6^	2.51 × 10^−4^	3.14 × 10^−2^

**Table 6:** Mathematical constants (A, B, C, D, and F) for HVL in the form HVL = A*x*^5^ + B*x*^4^ + C*x*^3^ + D*x*^2^ + E*x* + F (Equation (7)), determined from Figure 8.

Photon Energy (MeV)	Bi_2_O_3_					
	A	B	C	D	E	F
0.1	−5.83 × 10^−7^	7.00 × 10^−5^	−3.19 × 10^−3^	6.92 × 10^−2^	−7.46 × 10^−2^	3.92
0.5	0	0	0	0	−0.0854	6.796
1.0	0	0	0	0	−0.0878	9.469
5.0	0	0	0	0	−0.2492	21.923
**Photon Energy (MeV)**	**WO_3_**					
	**A**	**B**	**C**	**D**	**E**	**F**
0.1	−3.95 × 10^−7^	4.82 × 10^−5^	−2.25 × 10^−3^	5.13 × 10^−2^	−6.09 × 10^−2^	3.93
0.5	0	0	0	0	−0.0742	6.861
1.0	0	0	0	0	−0.0802	9.484
5.0	0	0	0	0	−0.2317	21.995
**Photon Energy (MeV)**	**BaSO_4_**					
	**A**	**B**	**C**	**D**	**E**	**F**
0.1	−7.63 × 10^−8^	1.01 × 10^−5^	−5.38 × 10^−4^	1.56 × 10^−2^	−2.93 × 10^−1^	3.93
0.5	0	0	0	0	−0.0546	6.917
1.0	0	0	0	0	−0.0665	9.494
5.0	0	0	0	0	−0.1919	22.093

**Table 7:** Pb_eq_ values of Bi_2_O_3_/PVA, WO_3_/PVA, and BaSO_4_/PVA hydrogels containing varying filler contents of 10, 20, 30, and 40 wt.%, determined at photon energies of 0.05, 0.08, and 0.1 MeV using XCOM.

Photon Energy (MeV)	Filler	Lead Equivalence (mmPb)				Recommended Content (wt.%)
		10 wt.%	20 wt.%	30 wt.%	40 wt.%	
0.05	Bi_2_O_3_	0.12	0.24	0.38	0.57	36
	WO_3_	0.08	0.16	0.25	0.36	--- *
	BaSO_4_	0.13	0.25	0.40	0.58	35
0.08	Bi_2_O_3_	0.16	0.28	0.42	0.61	34
	WO_3_	0.33	0.64	1.03	1.53	15
	BaSO_4_	0.17	0.28	0.43	0.60	34
0.1	Bi_2_O_3_	0.12	0.24	0.38	0.57	36
	WO_3_	0.09	0.17	0.27	0.39	---
	BaSO_4_	0.05	0.08	0.11	0.16	---

* --- implies the recommended content for the filler at the specific photon energy was beyond the range of 0–40 wt.%.
